# Effect of Gabapentin Pretreatment on Hyperalgesia in Older Patients Undergoing Sequential Bilateral Cataract Surgery: A Prospective, Randomized Controlled Trial

**DOI:** 10.3390/jcm15134944

**Published:** 2026-06-25

**Authors:** Seihee Min, Jiwon Han, Joo Youn Oh, Jeong-Hwa Seo

**Affiliations:** 1 Department of Anesthesiology and Pain Medicine, Asan Medical Center, University of Ulsan College of Medicine, Seoul 05505, Republic of Korea; seiheemin@gmail.com; 2 Department of Anesthesiology and Pain Medicine, Chung-Ang University Gwangmyeong Hospital, Chung-Ang University College of Medicine, Gwangmyeong 14353, Republic of Korea; yesuroon@gmail.com; 3 Department of Ophthalmology, Seoul National University Hospital, Seoul National University College of Medicine, Seoul 03080, Republic of Korea; bonzoo1@snu.ac.kr; 4 Department of Anesthesiology and Pain Medicine, Seoul National University Hospital, Seoul National University College of Medicine, Seoul 03080, Republic of Korea

**Keywords:** cataract, gabapentin, geriatric anesthesia, hyperalgesia, pain, postoperative, patient satisfaction

## Abstract

**Background/Objectives:** To evaluate whether gabapentin pretreatment modulates neuronal excitability and attenuates second-eye hyperalgesia in patients undergoing sequential bilateral cataract surgery under monitored anesthesia care with retrobulbar block. **Methods:** We conducted a prospective, parallel-group, randomized controlled trial with blinded outcome assessors at a tertiary university hospital. Patients aged > 60 years undergoing elective sequential bilateral cataract surgeries with a 1-week interval were enrolled. Participants were randomized 1:1 to receive gabapentin (100 mg three times daily for 1 week between surgeries) or no pretreatment. The primary outcome was the incidence of hyperalgesia, defined as greater intraoperative pain during the second-eye surgery compared to the first. Secondary outcomes included preoperative anxiety level, intraoperative and postoperative pain score, opioid rescue requirements, and surgeon/patient satisfaction for first- and second-eye surgeries. **Results:** Sixty-four patients (128 eyes) were included and analyzed (31 per group). Hyperalgesia was lower in the gabapentin pretreatment group than in the control group (4 [12.9%] vs. 17 [54.8%], risk ratio 0.24, 95% confidence interval 0.09–0.63, *p* = 0.001). Maximum intraoperative pain significantly decreased from the first to the second surgery in the gabapentin pretreatment group, whereas it increased in the control group (mean difference [standard deviation], −1.48 [2.14] vs. 0.97 [1.96], *p* < 0.001). Preoperative anxiety improved similarly in both groups before the second surgery. **Conclusions:** Gabapentin pretreatment may represent a promising strategy for reducing second-eye hyperalgesia and improving perioperative pain-related outcomes in older patients undergoing sequential bilateral cataract surgery under monitored anesthesia care with retrobulbar block.

## 1. Introduction

Cataract is commonly a bilateral ophthalmic condition and the world-leading cause of blindness, typically associated with the ageing process [1]. Surgical intervention remains the sole treatment for cataracts, consisting of extraction of the opacified lens followed by the implantation of a synthetic intraocular lens [2]. Sequential bilateral cataract surgery, usually performed at 1–2-week intervals, represents the current standard practice. This approach balances clinical safety, minimizing risks of bilateral endophthalmitis and refractive surprises while providing convenience for patients [3].

Recent advancements in surgical methodologies have established phacoemulsification as the prevailing technique, owing to its simplified procedure, reduced operation time, and promotion of accelerated functional recovery [4,5,6]. However, as phacoemulsification is not entirely pain-free, pain and anxiety remain significant concerns that can contribute to patient dissatisfaction [3,7]. Moreover, previous studies have demonstrated that the second-eye surgery results in more ocular pain than the first, despite identical surgical settings, anesthetic techniques, and surgical team [8,9,10]. This phenomenon may reflect tissue injury-induced hyperalgesia mediated by central sensitization mechanisms, whereby nociceptive pathways become hypersensitive following initial surgical trauma [3,9,11,12]. Nevertheless, evidence-based interventions targeting these perioperative concerns to improve patient satisfaction and recovery remain limited in the current literature.

Gabapentin is a widely used neuromodulator with established efficacy in neuropathic pain management and demonstrated anxiolytic properties in various anxiety disorders. By reducing neuronal excitability, gabapentin is particularly effective in the context of central sensitization [13,14].

This study aimed to investigate the effect of gabapentin pretreatment on pain and patient discomfort experienced during cataract surgery under monitored anesthesia care (MAC) with retrobulbar block. The primary outcome is the incidence of second-eye hyperalgesia, while secondary outcomes include preoperative anxiety scores derived from a self-assessment questionnaire [15], intraoperative and postoperative maximum pain experienced by patients, light sensitivity, intraoperative vital signs, and satisfaction levels of the surgeon and patients in the context of comparisons between the first- and second-eye surgeries.

## 2. Materials and Methods

### 2.1. Study Design and Ethical Approval

This prospective, parallel-group, randomized controlled trial was approved by the Institutional Review Board (approval number 1809-091-974, on 28 January 2019) and registered at ClinicalTrials.gov (protocol code NCT03826615, on 30 January 2019) prior to patient enrolment. The study was conducted in accordance with the Good Clinical Practice guidelines and the principles of the Declaration of Helsinki. All participants provided written informed consent after explanation of the nature and possible consequences of the study and retained the right to withdraw consent at any stage.

### 2.2. Study Population and Randomization

We enrolled patients aged > 60 years with American Society of Anesthesiologists (ASA) physical status I–III, scheduled for elective sequential bilateral cataract surgery with a 1-week interval under MAC. The exclusion criteria comprised simultaneous cataract surgery with other ophthalmic procedures, history of alcohol or drug abuse, diagnosis of mood disorders, consumption of central nervous system depressants or gabapentin, allergies to the study medications, renal impairment, body mass index < 16 kg/m^2^ or >30 kg/m^2^, pregnancy, or lack of consent to participate in the study.

After obtaining written informed consent, patients were randomized to either the gabapentin pretreatment or control group. An assistant not involved in the study randomly assigned groups in a 1:1 ratio, concealing the allocation sequence within sealed, opaque envelopes.

An unblinded investigator prescribed gabapentin (100 mg three times daily after meals) according to group assignment. The control group received no oral medication between surgeries. The interval between the first- and second-eye surgeries was 1 week for all enrolled participants. Gabapentin administration began immediately after the first-eye surgery and continued throughout this interval until the morning of the second-eye surgery. Because placebo medication was not administered, participant blinding could not be fully maintained. Nevertheless, to minimize expectation-related bias, participants were not informed of the specific study hypothesis regarding the potential effect of gabapentin on pain perception during second-eye surgery, and all outcome assessments were performed by investigators blinded to group allocation. On the first day of medication administration, the investigator conducted a follow-up phone call to assess adverse effects and patient discomfort. Patients were instructed to promptly report any unusual symptoms.

### 2.3. Study Protocol

The first- and second-eye surgeries were conducted under the same protocol. Patients were monitored with pulse oximetry, non-invasive blood pressure, heart rate, and electrocardiography. Before inducing MAC, an investigator who was blinded to group assignments assessed patients’ preoperative anxiety using the Amsterdam Preoperative Anxiety and Information Scale (APAIS) before admission to the operating room (Table 1). The APAIS questionnaire consists of six questions divided into three categories—surgery, anesthesia, and information—with two items in each category [13]. Upon arrival in the operating room, a retrobulbar block was performed by the ophthalmologist using a 27-gauge needle inserted via the inferolateral orbital approach. A mixture of lidocaine 2% (3–5 mL) and bupivacaine 0.5% (3–5 mL) was used to achieve anesthesia and akinesia. Following the retrobulbar block, MAC was induced and maintained using a target-controlled infusion of propofol (effect-site concentration, 1–3 µg/mL) and alfentanil (effect-site concentration, 1–3 ng/mL). Patients received supplemental oxygen at 3 L/min via nasal cannula, with end-tidal carbon dioxide monitoring performed by an unrelated anesthesiologist. Vital signs were monitored at 5 min intervals throughout the procedure, with additional measurements obtained immediately before and after phacoemulsification, known to be the surgical phase associated with the greatest nociceptive stimulation during cataract surgery. At the end of the surgery, maximum pain intensity experienced intraoperatively was evaluated using the visual analogue scale (VAS). Because the operated eye was covered immediately after surgery, patients used the non-operated eye to visualize the VAS chart. For individuals with insufficient visual acuity in the fellow eye, the blinded assessor verbally explained the anchors (“0 = no pain”, “10 = worst imaginable pain”) and recorded the patient’s numeric response. The degree of intraoperative light sensitivity caused by the surgical microscope was assessed using a 5-point scale (0, none; 1, minimal; 2, mild; 3, moderate; 4, severe).

Following surgery, postoperative pain was evaluated using VAS 20 min after arrival in the post-anesthesia care unit (PACU) and 1 h after transferring to the general ward. In PACU, fentanyl 50 µg was administered at the physician’s discretion according to the patient’s pain score. The surgeon assessed intraoperative satisfaction using a 3-point scale (−1 = dissatisfied; 0 = neutral; 1 = satisfied), evaluating patient cooperation based on observed movement, expressed pain, and anxiety during the procedure. Overall patient satisfaction with the surgical experience was assessed in the general ward using a 3-point scale (−1 = dissatisfied; 0 = neutral; 1 = satisfied).

### 2.4. Study Endpoints

The primary outcome of this study was the incidence of second-eye hyperalgesia, defined as greater intraoperative pain intensity during the second-eye surgery than during the first-eye surgery within the same patient, as assessed using the VAS. Secondary outcomes included the level of preoperative anxiety, assessed using the APAIS before the first and second surgeries, as well as the combined anxiety component score derived from four APAIS items to evaluate the overall level of worry and anxiety (Table 1). Additional secondary outcomes included the maximum pain and light sensitivity experienced during the first and second surgeries, intraoperative vital signs, postoperative pain measured at 20 min and 1 h after surgery, and overall satisfaction of both the surgeon and the patients.

### 2.5. Sample Size Calculation and Statistical Analysis

According to a previous study, 74% of patients undergoing LASIK surgery experienced increased pain during the second-eye operation compared to the first [16]. Given that cataract surgery is considered to involve a comparable procedural intensity and duration, we assumed a hyperalgesia incidence of 74% in the control group and hypothesized that gabapentin pretreatment would result in a 50% relative reduction in the incidence of hyperalgesia during the second-eye surgery. Based on the assumption, 28 patients were required for each group, achieving a power of 0.8 and an α of 0.05. Power analysis and group randomization were performed using STATA (Special Edition 14.2; Stata Corporation, College Station, TX, USA).

Continuous variables are presented as mean ± standard deviation (SD) or median (interquartile range, IQR) according to the normality of the variables. Categorical variables are expressed as numbers and percentages. A comparison of continuous variables between the control group and gabapentin group was conducted using independent two-sample t-tests or Wilcoxon rank-sum tests. Measurements at the first and second operations were compared using paired *t*-test or Wilcoxon signed-rank test. The association between categorical variables and treatment group was analyzed using Fisher’s exact test. A *p*-value of <0.05 was deemed statistically significant for all analyses. Analyses of secondary outcomes were considered exploratory, and no formal adjustment for multiple comparisons was made. R language version 4.4.1 (R Foundation for Statistical Computing, Vienna, Austria) and TnF program ver. 4.5 (YooJin BioSoft, Goyang-Si, Republic of Korea) were used for all the statistical analyses.

## 3. Results

**Figure 1:**
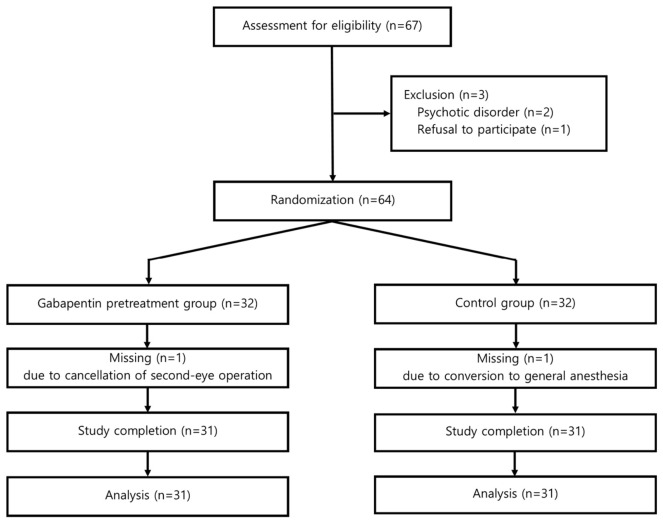
CONSORT diagram. CONSORT, Consolidated Standards of Reporting Trials.

Following the screening of 67 patients, 64 were randomly assigned to either the gabapentin pretreatment or the control group between 1 February 2019 and 3 January 2020. All participants assigned to the gabapentin group completed the prescribed 1-week treatment regimen. No treatment discontinuation related to adverse effects occurred during the study period, and no adverse events potentially attributable to gabapentin were observed. Two patients were excluded because of intraoperative conversion to general anesthesia and cancellation of the second-eye surgery (Figure 1), resulting in a total of 31 patients per group. No significant differences in patient characteristics were observed between the two groups (Table 2).

**Figure 2:**
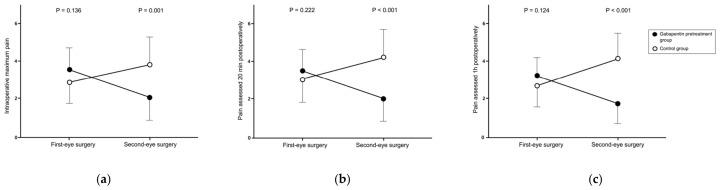
Comparison of pain scores assessed using VAS between first- and second-eye surgeries in each group. (**a**) Intraoperative maximum pain scores. (**b**) Pain assessed 20 min postoperatively. (**c**) Pain assessed 1 h postoperatively. VAS, visual analogue scale.

The primary outcome, the incidence of second-eye hyperalgesia, was significantly lower in the gabapentin pretreatment group than in the control group (4 [12.9%] vs. 17 [54.8%], risk ratio 0.24, 95% confidence interval 0.09–0.63, *p* = 0.001), corresponding to an absolute risk reduction of 41.9% and a number needed to treat of 3. The maximum intraoperative pain, evaluated using VAS, significantly decreased from the first to the second surgery in the gabapentin pretreatment group, whereas it significantly increased in the control group (mean difference ± SD, −1.48 ± 2.14 vs. 0.97 ± 1.96, *p* < 0.001) (Figure 2a). Gabapentin pretreatment and control groups displayed no significant differences in the amount of anesthetic agents administered before phacoemulsification or total amount of anesthetic administered during surgery across the first and second surgical procedures. Intraoperative mean blood pressure and heart rate also showed no significant differences between the first and second operations at baseline, immediately before, and after phacoemulsification in either group (Table 3).

**Figure 3:**
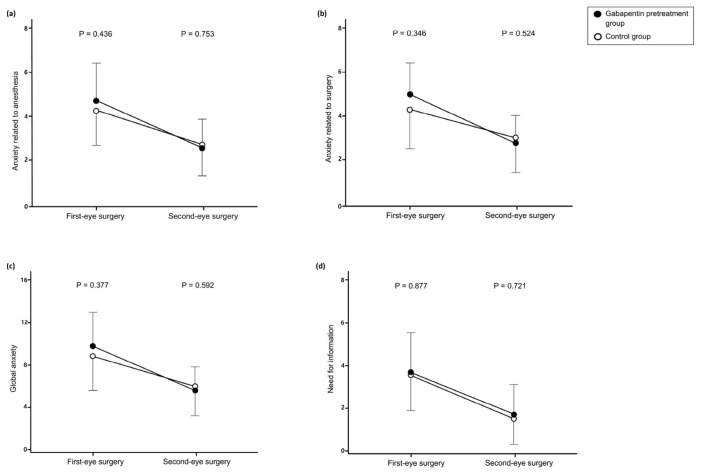
Comparison of APAIS scores between first- and second-eye surgeries per group. (**a**) Anxiety related to anesthesia, (**b**) anxiety related to surgery, (**c**) global anxiety, (**d**) need for information. APAIS, Amsterdam preoperative anxiety and information scale.

Patients’ preoperative anxiety levels, evaluated using the APAIS questionnaire, showed no significant differences in any of the four domains before the first or second surgeries between groups. Within-group analysis revealed that both groups demonstrated significantly reduced anxiety levels preceding the second surgery compared to the first surgery across all four domains (*p* < 0.05) (Figure 3).

Postoperative pain scores assessed in the PACU 20 min after surgery significantly increased from the first to the second surgery in the control group, whereas they significantly decreased in the gabapentin pretreatment group (1.23 ± 1.87 vs. −1.55 ± 2.35, *p* < 0.001) (Figure 2b). After the first surgery, fentanyl administration in the PACU was comparable between the groups (6 [19.4%] vs. 5 [16.1%], *p* = 1.000). However, following the second surgery, fentanyl administration was significantly higher in the control group than in the gabapentin pretreatment group (2 [6.5%] vs. 14 [45.2%], *p* = 0.001). Similarly, pain scores evaluated in the general ward 1 h after surgery showed a significant increase in the control group and a significant decrease in the gabapentin pretreatment group from the first to the second surgery (1.48 ± 1.98 vs. −1.48 ± 1.95, *p* < 0.001) (Figure 2c).

Regarding surgeon satisfaction, the number of cases rated as satisfactory in the control group decreased from 22 during the first-eye surgery to 17 during the second. In contrast, the gabapentin pretreatment group observed an increase in satisfactory cases from 19 during the first-eye surgery to 28 during the second. For patient satisfaction, the number of cases rated as “satisfactory” in the control group decreased from 19 during the first-eye surgery to 14 during the second surgery, whereas in the gabapentin pretreatment group, the number of satisfactory cases increased from 17 during the first-eye surgery to 24 during the second. Intraoperative light sensitivity did not demonstrate significant differences between the first and second surgeries in either group (*p* = 0.294 vs. 0.771).

## 4. Discussion

Cataract surgery is one of the most frequently performed procedures worldwide and has become a paradigmatic example of modern-day surgery [17,18]. Although cataract surgery has traditionally been regarded as a relatively pain-free procedure, recent studies have reported that patients may experience ocular pain during and after surgery [2,19,20,21]. Consequently, the procedure is performed under topical anesthesia in many countries; however, retrobulbar block remains widely practiced in several Asian regions. Patients undergoing sequential bilateral cataract surgery often report heightened pain during the second-eye surgery compared to the first, significantly influencing patient satisfaction and recovery [7,8,9,22,23]. This study suggests that gabapentin pretreatment may reduce second-eye hyperalgesia and improve perioperative pain-related outcomes in older patients undergoing sequential bilateral cataract surgery under MAC with retrobulbar block.

Cataract surgery, while minimally invasive, disrupts the blood–aqueous barrier and triggers local release of inflammatory cytokines in the operated eye, eliciting a sympathetic contralateral response following the initial procedure [3,12,22]. A recent systematic review reported that patients undergoing sequential bilateral cataract surgery under local anesthesia report greater pain during the second-eye surgery [10,17]. Likewise, a previous meta-analysis demonstrated that patients experience 1.5-fold greater pain during second-eye surgery compared to the first-eye surgery [9]. This clinical phenomenon, referred to as “second-eye syndrome”, may result from pathophysiological changes induced by central sensitization following the initial surgery, although its exact mechanisms remain unclear. Central sensitization is characterized by the hyperexcitability of dorsal horn neurons within the spinal cord, resulting in a decreased pain threshold to peripheral stimuli and heightening pain response. In the context of cataract surgery, tissue damage during the first-eye surgery may induce central sensitization through N-methyl-D-aspartate receptor activation at spinal level, leading to increased pain sensitivity during the second-eye surgery [3,9,12,24,25,26]. Another study observed a significant association between monocyte chemoattractant protein-1 levels in aqueous humor before the second-eye surgery and pain perception [12]. Consequently, patients experience greater ocular pain during second-eye surgery compared to the first, despite identical surgical conditions, anesthetic protocols, and the same surgical team.

Because intraoperative hyperalgesia may reduce patient cooperation, increase surgical difficulty, and diminish patient satisfaction, effective pain management should be considered an important component of care in bilateral sequential cataract surgery. With the growing emphasis on enhanced recovery after surgery (ERAS) principles, perioperative pain control and patient-centered outcomes have become increasingly important in ophthalmic surgery [15,27,28,29]. In the present study, gabapentin pretreatment significantly reduced the incidence of second-eye hyperalgesia and was associated with lower intraoperative and postoperative pain scores.

Gabapentin has demonstrated efficacy in managing peripheral neuropathic pain, postoperative pain, and particularly hyperalgesic conditions [30,31]. Chronic administration has been shown to attenuate central sensitization through binding to the α2δ subunit of voltage-dependent calcium channels and inhibiting central sensitization through interleukin-10 and heme oxygenase-1 pathways [13,14,32]. In addition, gabapentin possesses anxiolytic properties that have been reported in both perioperative and chronic pain settings [33,34]. Although pregabalin has pharmacokinetic advantages, gabapentin was selected because of its extensive clinical experience, established efficacy in central sensitization and perioperative hyperalgesia, and favorable safety profile in older surgical populations.

Owing to its favorable safety profile and potential benefits in pain modulation and anxiolysis, gabapentin may represent a useful component of multimodal perioperative pain management. Although the usual therapeutic dose range for neuropathic pain is higher, we selected a conservative regimen of 300 mg/day for several clinical reasons. First, the interval between the two surgeries was limited to 1 week, restricting the feasibility of gradual dose escalation. Second, the study population consisted exclusively of older adults, in whom dose-related adverse effects such as somnolence, dizziness, gait instability, and cognitive impairment are clinically relevant concerns, particularly in an ambulatory surgical setting. Third, our objective was to attenuate evolving central sensitization following first-eye surgery rather than to treat established chronic neuropathic pain. Therefore, a low-dose regimen was considered appropriate to balance potential neuromodulatory effects with safety and tolerability. The absence of treatment discontinuation or gabapentin-related adverse events in the present study further supports the feasibility of this approach, although future studies should explore potential dose–response relationships.

Consequently, patients in the gabapentin pretreatment group demonstrated significantly reduced pain during second-eye surgery compared with first-eye surgery, whereas pain increased in the control group. These findings suggest that gabapentin pretreatment may attenuate central sensitization and thereby reduce the incidence of second-eye hyperalgesia. In addition, the reduced requirement for rescue opioids in the PACU suggests a clinically meaningful analgesic benefit. Although both groups demonstrated lower anxiety scores before second-eye surgery, the between-group difference did not reach statistical significance.

This study has some limitations. First, the relatively small sample size may limit the generalizability of our findings. Second, the absence of a placebo control and the inability to maintain formal participant blinding represent important methodological limitations. Because the primary outcome and several secondary outcomes, including pain scores and patient satisfaction, were subjective, they may have been influenced by expectation or placebo-related effects. To mitigate this risk, participants were not informed of the specific study hypothesis regarding second-eye pain modulation, outcome assessors remained blinded to treatment allocation, and objective or assessor-rated outcomes, including postoperative opioid requirements in the PACU and surgeon-rated intraoperative cooperation, were consistent with the observed analgesic effect. Nevertheless, expectation bias cannot be completely excluded, and the findings should therefore be confirmed in adequately powered, double-blind, placebo-controlled trials. Third, retrobulbar anesthesia rather than topical anesthesia was used, which may have reduced the absolute nociceptive input. However, relative differences between first- and second-eye surgeries remained evident, consistent with the hypothesis of central sensitization. Fourth, dose-dependent effects of gabapentin were not evaluated. In addition, because this study was conducted at a single tertiary care center and included only patients undergoing cataract surgery under MAC with retrobulbar block, the findings should not be generalized to all cataract surgery settings. Further studies are needed to determine whether similar effects can be observed in patients undergoing surgery under topical or intracameral anesthesia alone.

In conclusion, gabapentin pretreatment may represent a promising strategy for reducing second-eye hyperalgesia and improving perioperative pain-related outcomes in older patients undergoing sequential bilateral cataract surgery under MAC with retrobulbar block. Larger placebo-controlled studies are warranted to confirm these findings and determine their applicability across different cataract surgery settings.

## Figures and Tables

**Table 1:** Amsterdam Preoperative Anxiety and Information Scale (APAIS).

Agreement with each statement should be graded on a five-point Likert scale from 1 = not at all to 5 = extremely
A1 I am worried about the anesthetic.
A2 The anesthetic is on my mind continually.
A3 I would like to know as much as possible about the anesthetic.
C1 I am worried about the procedure.
C2 The procedure is on my mind continually.
C3 I would like to know as much as possible about the procedure.
Average scores calculated from the Amsterdam scale
A1 + A2: Anxiety related to anesthesia
C1 + C2: Anxiety related to surgery
A1 + A2 + C1 + C2: Global anxiety
A3 + C3: Need for information

**Table 2:** Patient characteristics.

Variables	Gabapentin Pretreatment Group (*n* = 31)	Control Group (*n* = 31)
Age (years)	71.42 (7.98)	72.81 (6.18)
Female	20 (64.5%)	27 (87.1%)
Height (cm)	156.06 (9.38)	154.03 (6.62)
Body Mass Index (kg m^−2^)	24.35 (3.23)	23.83 (2.87)
ASA physical status (I/II/III)	4/27/0	0/28/3
Underlying disease		
(hypertension/diabetes/stroke)	20/10/2	11/10/3
Cataract etiology		
(senile/pathological/iatrogenic)	29/2/0	28/3/0
Cataract type		
(cortical/nuclear/cortical + nuclear/posterior subcapsular)	9/3/11/8	8/5/6/12

Data are number of patients or mean (SD). There are no differences between groups. ASA, American Society of Anesthesiologists; SD, standard deviation.

**Table 3:** Comparison of anesthetic and surgical parameters between first- and second-eye surgeries.

Variables	Group	First Surgery	Second Surgery	Difference	*p*-Value
Duration of surgery (min)	Gabapentin pretreatment	18.32 (6.31)	16.97 (5.40)	−1.35 (7.05)	0.293
	Control	21.55 (8.98)	20.39 (6.05)	–1.16 (8.12)	0.432
Duration of anesthesia (min)	Gabapentin pretreatment	28.32 (6.74)	27.35 (6.32)	–0.97 (7.64)	0.486
	Control	31.61 (9.34)	28.68 (6.32)	–2.94 (8.19)	0.055
**Cumulative doses administered before phacoemulsification**					
Propofol (mg)	Gabapentin pretreatment	22.88 (5.02)	23.74 (6.36)	0.86 (7.09)	0.502
	Control	19.65 (7.41)	19.61 (6.29)	–0.03 (7.30)	0.981
Alfentanil (μg)	Gabapentin pretreatment	286.77 (63.42)	296.77 (79.51)	10.00 (90.00)	0.541
	Control	250.65 (91.10)	250.32 (77.52)	–0.32 (91.16)	0.984
**Total doses administered during the operation**					
Propofol (mg)	Gabapentin pretreatment	33.29 (5.50)	33.29 (6.56)	0.00 (7.73)	>0.99
	Control	32.06 (7.31)	31.23 (5.70)	–0.84 (7.48)	0.537
Alfentanil (μg)	Gabapentin pretreatment	416.13 (68.78)	416.13 (82.04)	0.00 (96.61)	>0.99
	Control	400.65 (91.72)	390.32 (71.20)	–10.32 (93.83)	0.545
**Intraoperative hemodynamic variables**					
Baseline HR	Gabapentin pretreatment	65.35 (11.90)	64.58 (9.63)	–0.77 (8.22)	0.604
	Control	70.35 (12.34)	70.29 (12.85)	–0.06 (6.77)	0.958
Baseline MBP (mmHg)	Gabapentin pretreatment	100.55 (17.29)	98.35 (13.29)	–2.19 (12.33)	0.330
	Control	110.23 (14.87)	110.23 (14.66)	0.00 (15.51)	>0.99
HR before phacoemulsification	Gabapentin pretreatment	63.58 (9.55)	63.19 (10.60)	–0.39 (6.64)	0.748
	Control	70.42 (13.61)	69.84 (12.26)	–0.58 (6.48)	0.622
MBP before phacoemulsification (mmHg)	Gabapentin pretreatment	93.06 (10.42)	89.84 (13.03)	–3.23 (10.25)	0.090
	Control	103.32 (15.67)	99.84 (12.59)	–3.48 (16.46)	0.248
HR after phacoemulsification	Gabapentin pretreatment	63.00 (9.84)	62.52 (9.98)	–0.48 (5.86)	0.649
	Control	69.68 (13.06)	68.81 (12.41)	–0.87 (6.03)	0.428
MBP after phacoemulsification (mmHg)	Gabapentin pretreatment	91.42 (10.60)	88.94 (13.42)	–2.48 (11.35)	0.232
	Control	101.19 (13.93)	98.13 (14.72)	–3.06 (18.58)	0.366

Data are presented as mean (SD). HR, heart rate; MBP, mean blood pressure; SD, standard deviation.

## Data Availability

The data that support the findings of this study are available from the corresponding author upon reasonable request.

## Abbreviations

The following abbreviations are used in this manuscript:

MAC	Monitored anesthesia care
ASA	American Society of Anesthesiologists
APAIS	Amsterdam Preoperative Anxiety and Information Scale
VAS	Visual analogue scale
PACU	Post-anesthesia care unit
SD	Standard deviation
IQR	Interquartile range
ERAS	Enhanced recovery after surgery
